# Serous Ovarian Cancer Following Opportunistic Bilateral Salpingectomy

**DOI:** 10.1001/jamanetworkopen.2025.57267

**Published:** 2026-02-02

**Authors:** Ramlogan Sowamber, Alice J. Mei, Paramdeep Kaur, Julianne McLeod, Emily McKay, Alex Lukey, Jamie Bakkum-Gamez, Natalia Buza, Paul A. Cohen, Kyle Devins, Rhonda Farrell, Christine Garcia, Blake Gilks, Ellen Goode, Anjelica Hodgson, Brooke Howitt, Pei Hui, Jutta Huvila, Anthony Karnezis, Kianoosh Keyhanian, Mary Kinloch, Martin Köbel, Felix K. F. Kommoss, Lawrence Kushi, Janice S. Kwon, Kara Long-Roche, Anais Malpica, Jessica N. McAlpine, Dianne Miller, Esther Oliva, Andrea Palicelli, Aleksandra Paliga, Carlos Parra-Herran, Celeste Leigh Pearce, Sharnel Perera, Jurgen M. Piek, Haiyan Qiu, Joseph Rabban, Robert Rome, Miranda Steenbeek, Rebecca Stone, Aline Talhouk, Kristin M. Tischer, Britton Trabert, Penelope M. Webb, John R. Zalcberg, David G. Huntsman, Gillian E. Hanley

**Affiliations:** 1 University of British Columbia, Vancouver, British Columbia, Canada; 2 Mayo Clinic, Rochester, Minnesota; 3 Yale School of Medicine, New Haven, Connecticut; 4 University of Western Australia, Perth, Western Australia, Australia; 5 Harvard Massachusetts General Hospital, Boston; 6 Chris O'Brien Lifehouse, Camperdown, Sydney, New South Wales, Australia; 7 Kaiser Permanente Northern California, Pleasanton; 8 University Health Network, Toronto, Ontario, Canada; 9 Stanford University, Stanford, California; 10 University of Turku, Turku, Finland; 11 University of California Davis, Sacramento; 12 University of Ottawa, Ottawa, Ontario, Canada; 13 University of Saskatchewan, Saskatoon, Slovakia, Canada; 14 University of Calgary, Calgary, Alberta, Canada; 15 University Hospital Heidelberg, Heidelberg, Baden-Württemberg, Germany; 16 Memorial Sloan Kettering, New York, New York; 17 University of Texas, Austin; 18 Azienda USL-IRCCS di Reggio Emilia, Reggio Emilia, Italy; 19 Harvard Brigham and Women's Hospital, Boston, Massachusetts; 20 University of Michigan School of Public Health, Ann Arbor; 21 Monash University, Melbourne, Victoria, Australia; 22 Catharina Cancer Institute and Radboud University Medical Center, Nijmegen, the Netherlands, Catharina Ziekenhuis, Eindhoven, the Netherlands; 23 University of California San Francisco; 24 University of Melbourne, Melbourne, Victoria, Australia; 25 Johns Hopkins Medicine, Baltimore, Maryland; 26 Huntsman Cancer Institute at the University of Utah, Salt Lake City; 27 QIMR Berghofer Medical Research Institute, Brisbane, Queensland, Australia; 28 Alfred Health, Melbourne, Victoria, Australia

## Abstract

This cohort study evaluates the reduction in risk of high-grade serous carcinoma among women undergoing opportunistic bilateral salpingectomy in Canada.

## Introduction

Ovarian carcinoma is a heterogeneous disease with a 5-year survival rate below 50%.^1^ Primary prevention of the most common histotype of ovarian carcinoma (high-grade serous carcinoma [HGSC], 70% of ovarian carcinomas) is possible using opportunistic bilateral salpingectomy (OBS; the removal of the fallopian tubes during another pelvic surgery while conserving the ovaries). Significant data show that OBS is safe,^2^ it does not appear to reduce the age of onset of menopause,^3^ and it is cost-effective.^4^ Herein, this evidence base is expanded by (1) estimating the risk reduction for serous ovarian cancer afforded by OBS using population-based data; and (2) examining whether the histotype distribution of ovarian carcinomas in people without fallopian tubes significantly differs from the historical histotype distribution.

## Methods

For the first aim, we conducted a population-based retrospective cohort study including all people who underwent a hysterectomy or tubal permanent contraception in British Columbia between 2008 and 2020 (eTable 1 and eTable 2 in Supplement 1), with approval from the University of British Columbia’s clinical research ethics board and a waiver of consent due to use of deidentified data. We followed Strengthening the Reporting of Observational Studies in Epidemiology (STROBE) reporting guidelines. Risk reduction for serous carcinomas from the population-based data was estimated using Cox proportional hazards models comparing individuals in the OBS group with individuals in the comparison surgery group (hysterectomy alone or tubal ligation). Low- and high-grade serous carcinomas were combined as our data did not specify histotype among serous carcinomas, but HGSC represent 95% of these carcinomas. We repeated the analysis with breast cancer as the outcome to examine the likelihood of selection bias on important unmeasurable differences between the groups (see eMethods in Supplement 1).

For the second aim, we asked international pathologists to enter anonymized data into a RedCap database for any ovarian carcinomas occurring in a patient without fallopian tubes (eTable 2 in Supplement 1). The histotype distribution of these cancers was compared with a historical histotype distribution using Fisher exact test.^5^ All *P* values were 2-sided, and statistical significance was defined as *P* < .05 for all analyses, which were performed in SAS version 9.4 (SAS Institute) and Stata version 19 (StataCorp) from April 2025 to October 2025.

## Results

In aim 1, there were 85 823 patients who had surgical procedures of interest, 40 527 who underwent OBS (median [IQR] follow-up, 4.72 [2.23-7.09] years), and 45 296 who underwent a comparator surgery (median [IQR] follow-up, 8.45 [6.07-11.51] years). Compared with those who underwent OBS, individuals in the comparison group were older at the time of surgery (mean [SD] age, 42.4 [12.6] vs 40.7 [8.1] years) and had less use of oral contraceptive pills (21 665 individuals [50.0%] vs 23 876 [60.7%]; mean [SD] use, 1322 [1465] vs 1085 [1230] days) (Table). The crude hazard ratio for serous ovarian carcinoma was 0.22 (95% CI, 0.05-0.95); for breast cancer, it was 0.99 (95% CI, 0.84-1.17). For aim 2, 26 ovarian carcinomas were identified in individuals without fallopian tubes, with only 6 of 26 (23.1%) being HGSC compared with 642 of 942 (68.1%) in a historical cohort with fallopian tubes (Fisher exact test, *P* < .001) (Figure).^5^

**Table.** Comparison of Important Risk and Protective Factors for Ovarian Cancer Between the Opportunistic Bilateral Salpingectomy (OBS) Group and Comparator Surgery Group

Factor	Participants, No. (%)		SMD
	Comparator surgery (n = 45 296)	OBS (n = 40 527)	
Age at surgery, mean (SD)	42.4 (12.6)	40.7 (8.1)	0.16
Follow-up, median (IQR), y	8.45 (6.07-11.51)	4.72 (2.23-7.09)	1.11
Income quintile^a^			
1 (lowest 20%)	8709 (20.1)	7191 (18.3)	0.07
2	9163 (21.1)	7856 (20.0)	
3	8784 (20.3)	7981 (20.3)	
4	8591 (19.8)	8416 (21.4)	
5 (highest 20%)	7470 (17.2)	7533 (19.2)	
Missing	656 (1.2)	354 (0.90)	
Parity, live births, mean (SD)	1.98 (1.1)	1.91 (1.0)	0.06
No. of pregnancies, mean (SD)	2.41 (1.5)	2.32 (1.4)	0.07
OCP use	21 665 (50.0)	23 876 (60.7)	0.22
OCP duration, mean (SD), days^b^	1085 (1230)	1322 (1465)	0.18
Endometriosis	4460 (9.9)	5251 (13.0)	0.09
Serous ovarian cancer^c^			
No. of person years	370 133	189 101	NA
Cancer events	21	≤5^d^	NA
Breast^e^			
No. of person years	368 138	188 418	NA
Cancer events	492	218	NA

Abbreviations: OCP, oral contraceptive pill; NA, not applicable; SMD, standardized mean difference.

^a^ Based on census-based province-wide distribution.

^b^ Among OCP users.

^c^ Hazard ratio, 0.22 (95% CI, 0.05-0.95).

^d^ Cell sizes 1 through 5 are suppressed according to privacy requirements of the data stewards. These numbers must be reported as ≤5.

^e^ Hazard ratio, 0.99 (95% CI, 0.84-1.17).

**Figure.**
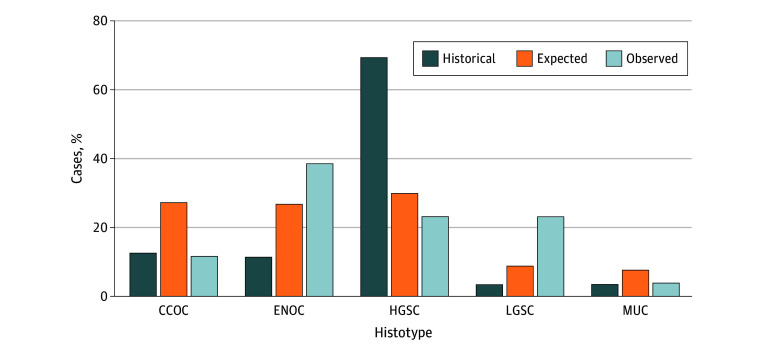
Histotype Distribution Following a Bilateral Salpingectomy in the Cases Entered Into the RedCap Database The dark blue bars represent the historical distribution of ovarian cancers in women who have not had an opportunistic bilateral salpingectomy, as determined by Kobel et al.^5^ The orange bars represent the expected numbers of ovarian cancers following a hypothetical application of an 80% reduction to the high-grade serous carcinoma histotype. The light blue bars indicate the actual observed ovarian cancers from patients who had undergone bilateral salpingectomy. The high-grade serous carcinoma (HGSC) histotype was significantly decreased in the observed cohort (23.1% of ovarian cancer cases) following bilateral salpingectomy compared with the historical histotype distribution of HGSC (68.1% of all ovarian carcinoma cases). Fisher exact test, *P* < .001. CCOC indicates clear cell ovarian carcinoma; ENOC, endometrioid ovarian carcinoma; LGSC, low-grade serous carcinoma; MUC, mucinous ovarian cancer.

## Discussion

In this expanded analysis of health care and cancer data from British Columbia, we add to preliminary evidence of effectiveness of OBS^6^ as individuals who underwent OBS were at nearly 80% reduced risk for serous ovarian cancers compared with those who had a hysterectomy alone or tubal ligation. Our second aim showed significantly fewer HGSCs in people without fallopian tubes compared with the historical histotype distribution for ovarian carcinoma.

Limitations include that many of these surgical procedures occurred in people well below the age of peak risk for HGSC, which resulted in a small number of ovarian carcinomas. This meant we could not control for all possible confounders in the Cox proportional hazards models. Nonetheless, these findings provide robust support for the effectiveness of OBS as a preventive intervention and underscore that broader implementation of OBS has the potential to significantly reduce the incidence and mortality of serous ovarian carcinoma.

## Notes

Supplement 1. eMethods. eTable 1. Details on the Data Used for the Population-Based Cohort eTable 2. Data Dictionary for Population-Based Cohort eReferences.

Supplement 2. Data Sharing Statement
