# Supplementary material for: Serous Ovarian Cancer Following Opportunistic Bilateral Salpingectomy
Source: JAMA Netw Open. 2026 Feb 2;9(2):e2557267. doi: 10.1001/jamanetworkopen.2025.57267 (PMC12865655; doi:10.1001/jamanetworkopen.2025.57267)
Supplement: Supplement 1. — eMethods. eTable 1. Details on the Data Used for the Population-Based Cohort eTable 2. Data Dictionary for Population-Based Cohort eReferences. [file jamanetwopen-e2557267-s001.pdf]

Supplemental Online Content

Sowamber R, Mei AJ, Kaur P, et al. Serous ovarian cancer prevention with opportunistic bilateral salpingectomy. *JAMA Netw Open*. 2026;9(2):e2557267.
doi:10.1001/jamanetworkopen.2025.57267

eMethods.
eTable1. Details on the Data Used for the Population-Based Cohort
eTable2. Data Dictionary for Population-Based Cohort
eReferences.

This supplemental material has been provided by the authors to give readers additional information about their work.

© 2026 Sowamber R et al. *JAMA Network Open*.

eMethods.

Aim 1: Population-based data

Inclusion criteria

Identify all females undergoing the following procedure of interest:

1. Hysterectomy
2. Salpingectomy
3. Tubal ligation

We defined the years that are included based on when OS became common practice in each particular geographic region.

Exclusion Criteria

1. Those with a diagnosis of ovarian cancer before or within 6 months of their surgery as this predated the surgery
2. Those who underwent an oophorectomy before or during the procedure of interest
3. Those with diagnostic codes indicative of gynecologic cancer at the time of their surgery (ovary, cervix, endometrial, vaginal/vulvar)

Identification of the Exposure Group

Individuals who underwent any relevant surgical procedure were identified using the Canadian Classification of Health Intervention codes. Those with a diagnosis of any gynecologic cancer before or within six months of their surgery were excluded, as these cancers were likely present at the time of surgery. Individuals were stratified into two groups according to their procedures: 1) those who underwent OBS, meaning they had a hysterectomy (CCI procedure code 1.RM.89.X) with a bilateral salpingectomy (CCI 1.RF.89.X) but no oophorectomy (CCI 1.RB.89.X or 1.RD.89.X), or individuals who had a bilateral salpingectomy alone with a diagnosis code indicating the procedure was for sterilization (ICD-10-CM Z30.2), which could have occurred at the time of C-section or interval sterilizations; and, 2) comparator surgeries, which included individuals who had undergone a hysterectomy with no concomitant oophorectomy or salpingectomy and anyone who underwent a tubal ligation (CCI 1.RF.51.X).

Identification of the Outcome

Provincial registries include cases identified through pathology reports, death certificates, electronic medical records, and cancer treatments. All ovarian cancers were identified using the *International Classification of Diseases for Oncology codes.* We included ovarian cancer, fallopian tube cancer or peritoneal cancer, not otherwise specified (C56.X; C57.X; C48.2, respectively). Using an algorithm published by Peres et al.[1] and ICD-O morphology codes, the histotype of ovarian carcinomas was extracted by searching all invasive EOCs diagnosed following a surgery of interest.[2] We excluded serous tubal intraepithelial neoplasm. Since information on tumor grade was incomplete in earlier years, we had to group high-grade and low-grade serous cancers into a single serous group.

Examining potential measured and unmeasured confounding

We compared the following known and measured risk and protective factors for ovarian cancer between the OBS group to the comparison surgery group: age at time of surgery, income quintile, parity, gravidity, history of oral contraceptive pill use, total mean days of oral contraceptive use, and presence of endometriosis (ICD 10 CA N80.X) listed as an indication or contributing condition to their surgical episode at OBS or their comparison surgery. To examine whether our results could have been biased by unmeasured confounding factors such as genetic differences, lifestyle differences or degree of investment in cancer prevention, we conducted the same analysis examining breast cancer as the outcome, as OBS should not affect risk for breast cancer and thus any difference between groups would be indicative of important group differences biasing the results.

© 2026 Sowamber R et al. *JAMA Network Open*.

Population-based data approvals

Approvals were obtained from all relevant data stewards and access to the Consolidation file, the BC Cancer Registry, the Discharge Abstract Database, and PharmaNet was facilitated through Population Data BC.

Aim 2: Histotype distribution of EOCs in people without fallopian tubes

An international group of collaborators searched retrospectively through pathology reports and clinical databases to identify cases, defined as ovarian carcinomas in patients who had a bilateral salpingectomy without a bilateral oophorectomy. Histotype pathology was reviewed by gynecological pathologists and data acquisition requests were performed securely and in compliance with privacy guidelines set out by each institution (UBC REB:H22-00796). Study data were collected and managed using REDCap (securely hosted at the University of British Columbia). Inclusion criteria considered all females from the general population with no known pathogenic variant and no previous indication of an ovarian cancer. Exclusion criteria were limited to individuals who had been diagnosed with ovarian cancer before or within six months of surgery; who underwent bilateral oophorectomy before or during the surgery of interest; and who had gynecologic cancer (ovary, cervix, endometrial, vaginal/vulvar) at the time of surgery. Subjects with a unilateral salpingectomy were also excluded, but subjects with a unilateral oophorectomy and a bilateral salpingectomy were included. We compared the historical histotype distribution for invasive EOC with an expected histotype distribution that assumed an 80% reduction of HGSC, which was proposed based on previous studies that investigated a surgery and ovarian cancer risk reduction.[3] This assumption shows the EOC cases that could be prevented and the residual cases that would remain once the prevented cases were removed from the population.

Statistical Analyses

Aim 1: Censoring for the Cox Proportional hazards models occurred at the event (EOC), death, moving out of the province or at the end of the study period (December 31, 2020). Given the low number of ovarian cancers in the OBS group, we could not control for confounding variables. Instead, we compared the potential confounders across the OBS and comparator surgery group and calculated their standardized mean differences. A difference between covariates was considered meaningful if the standardized mean difference was greater than 0.1.

Aim 2: The histotype proportion in the case series was compared to the histotype proportion in a historical ovarian cancer cohort of patients. The historical cancer cohort was based on reviewing slide sets of 1009 cases of 2555 patients diagnosed with ovarian carcinoma that were referred to the British Columbia Cancer Agency over a 16-year period (1984 to 2000), which showed that the overall frequency of tumor types was as follows: high-grade serous—68.1%, clear-cell—12.2%, endometrioid—11.3%, mucinous—3.4%, low-grade serous—3.4%, rare types—1.6%. This distribution was then validated using 410 consecutive cases from the Washington Hospital Center and found to hold in that setting.[4] Thus, we used the histotype distributions reported in that analysis to compare to our case series using a Fisher's Exact test. Non-epithelial ovarian cancers were identified, but not included in statistical analyses as OBS is designed to prevent epithelial ovarian cancers, and because expected numbers of cancers for these non-epithelial ovarian cancers could not be identified from literature. All p-values were two-sided and statistical significance was defined as $p<0.05$ for all analyses (performed in SAS and STATA)

Statistical software and packages

SAS

The output and data analysis for this paper was generated using SAS 9.4 software. Copyright © 2024. SAS Institute Inc. SAS and all other SAS Institute Inc. product or service names are registered trademarks or trademarks of SAS Institute Inc., Cary, NC, USA.

STATA

StataCorp. (2025). Stata Statistical Software: Release 19. College Station, TX: StataCorp LLC.

R studio

Posit team. (2025). RStudio: Integrated Development Environment for R. Posit Software, PBC, Boston, MA. URL http://www.posit.co/.

R packages

R Core Team. (2024). R: A Language and Environment for Statistical Computing. R Foundation for Statistical Computing, Vienna, Austria, https://www.R-project.org/.

Wickham H, Averick M, Bryan J, Chang W, McGowan LD, François R, Grolemund G, Hayes A, Henry L, Hester J, Kuhn M, Pedersen TL, Miller E, Bache SM, Müller K, Ooms J, Robinson D, Seidel DP Spinu V, Takahashi K, Vaughan D, Wilke C, Woo K, Yutani H (2019). Welcome to the tidyverse. Journal of Open Source Software, 4(43), 1686.doi:10.21105/joss.01686 https://doi.org/10.21105/joss.01686

H. Wickham. (2016). ggplot2: Elegant Graphics for Data Analysis. Springer-Verlag New York.

Wickham H, Bryan J. (2025). readxl: Read Excel Files. R package version 1.4.5, https://CRAN.R project.org/package=readxl.

Rich B. (2023).Table1: Tables of Descriptive Statistics in HTML. R package version 1.4.3, https://CRAN.R project.org/package=table1.

Iannone R, Cheng J, Schloerke B, Hughes E, Lauer A, Seo J, Brevoort K, Roy O. (2025). _gt: Easily Create Presentation-Ready Display Tables_. R package version 1.0.0, https://CRAN.R project.org/package=gt.

© 2026 Sowamber R et al. *JAMA Network Open*.

eTable1. Details on the Data Used for the Population-Based Cohort

Dataset	Data contained in dataset	Years
Consolidation File	BC health insurance registration information for the entire population of the province	2008-2020
Discharge Abstract Database[2]	All hospital stays and day surgeries performed in the province<br>• Surgical procedures undertaken<br>• ICD-10 diagnostic codes	2008-2020
BC Cancer Registry[3]	All cancer diagnosed in the province<br>• ICD for Oncology Diagnostic codes<br>• ICD-O morphology codes<br>• Date of diagnosis	2008-2020

© 2026 Sowamber R et al. *JAMA Network Open*.

eTable2. Data Dictionary for Population-Based Cohort

STUDY IDs	Text/Num	Notes
Study ID	Num	**Info:** A unique study specific ID applied to each person in the study that is linkable across different datasets
**Surgical data**	**Text/Num**	**Notes**
Procedure codes	Char	**Additional Info:** Codes for identifying which procedure or combination of procedures were undertaken in the surgery
Laterality	Char	**Additional Info:** Codes to identify whether the person had both (=B), the right (=R) the left (=L) or unknown number of body parts removed. This is important for ensuring that bilateral salpingectomy was undertaken.
Year of surgery	Num	**Additional info:** Codes to capture appropriate inclusion years and to control for temporality.
Age at surgery	Num	**Additional info**: Codes for month and year of birth.
Other benign gynecologic conditions coded at surgery	Char	**Additional info**: In BC, ICD10 is used. Conditions like endometriosis, leiomyoma, benign ovarian or uterine neoplasm, abnormal bleeding, pelvic organ prolapse, pelvic inflammatory disease, hydrosalpinx are captured.
Surgical approach	Char	**Additional info:** Code indicating whether the surgery was open, laparoscopic, vaginal, combination or robotic.
Group	Num	**Additional info:** Codes for hysterectomy with OS; hysterectomy alone (ovarian conservation without fallopian tube removal; tubal ligation and OS for sterilization groups.<br>*Hysterectomy with OS*: Code for hysterectomy with code for salpingectomy and code indicating it was bilateral.<br>*Hysterectomy alone*: Code only for hysterectomy<br>*Salpingectomy for sterilizati*on: Code for salpingectomy with diagnostic code indicating it was for sterilization. If the sterilization code is not used in all datasets, then code indicating the salpingectomy was bilateral should be sufficient.<br>*Tubal ligation*: Separate code captured in this field.
**Demographic Info and Covariates**	**Text/Num**	**Notes**
Geographic Income Band (for year of birth)	Num	**Source:** Statistics Canada<br><br>**Additional Info:** The Income Band data file consists of 1000 income bands of data summarizing equivalized disposable income, derived from Statistics Canada tax-filer data prepared for the years 1992, 2002, and

© 2026 Sowamber R et al. *JAMA Network Open*.

		2006. All income bands are made up of an entire postal code. Equivalized disposable income for each postal code is calculated and averaged.
Income quintiles	Num	**Additional Info**: Neighborhood based ranking of income. Any measure of SES is captured.
Urban/Rural Status	Char	**Additional info**: Local health area, where patients reside are used to classify people into urban, rural, remote.
Parity	Num	**Source:** Vital Stats BC birth certificate data<br>**Additional info:** Number of live births from our vital statistics data is captured in this field.
Previous years of oral contraceptive pill use	Num	**Source:** BC PharmaNet (prescription dispensation for everyone in province)
**Ovarian cancer diagnosis-Outcome**	**Text/Num**	**Notes**
ICD 10 code	Char	**Additional info:** Code C56.X, C57.X and C48.2 was used.
Histology code	Char	**Additional info:** Codes captured through algorithm published by Peres et al.
Date of diagnosis	Date	**Additional info:** Field captures dates.

eReferences

1. Peres LC, Cushing-Haugen KL, Köbel M, et al. Invasive Epithelial Ovarian Cancer Survival by Histotype and Disease Stage. J Natl Cancer Inst 2019;111:60-8.
2. Hanley GE, Pearce CL, Talhouk A, et al. Outcomes From Opportunistic Salpingectomy for Ovarian Cancer Prevention. JAMA Netw Open 2022;5:e2147343.
3. Finch A, Beiner M, Lubinski J, et al. Salpingo-oophorectomy and the Risk of Ovarian, Fallopian Tube, and Peritoneal Cancers in Women With a BRCA1 or BRCA2 Mutation. JAMA 2006;296:185-92.
4. Kobel M, Kalloger SE, Huntsman DG, et al. Differences in tumor type in low-stage versus high-stage ovarian carcinomas. Int J Gynecol Pathol 2010;29:203-11.

© 2026 Sowamber R et al. *JAMA Network Open*.
