# Supplementary material for: Serous Ovarian Cancer Following Opportunistic Bilateral Salpingectomy
Source: JAMA Netw Open. 2026 Feb 2;9(2):e2557267. doi: 10.1001/jamanetworkopen.2025.57267 (PMC12865655; doi:10.1001/jamanetworkopen.2025.57267)
Supplement: Supplement 2. — Data Sharing Statement [file jamanetwopen-e2557267-s002.pdf]

Data Sharing Statement

Sowamber. Serous Ovarian Cancer Prevention with Opportunistic Bilateral Salpingectomy. *JAMA Netw Open*. Published February 02, 2026. doi:10.1001/jamanetworkopen.2025.57267

Data
Data available: Yes, the researcher-collected data for aim 2 is available. Data from aim 1 is available is researchers apply for data access; more details available at https://www.popdata.bc.ca/
Data types: Deidentified participant data, Data dictionary for aim 2
How to access data: dhuntsma@bccancer.bc.ca
When available: With publication

Supporting Documents
Document types: None

Additional Information
Who can access the data: Researchers whose proposed use of the data has been approved.
Types of analyses: For any purpose.
Mechanisms of data availability: After approval of a proposal and with investigator support.
